# Oxytocin is needed, but it is no magic bullet

**DOI:** 10.1111/1471-0528.14134

**Published:** 2016-06-06

**Authors:** AD Weeks

**Affiliations:** ^1^ Sanyu Research UnitDepartment of Women's and Children's HealthUniversity of LiverpoolLiverpoolUK

Uterotonic therapy is critical in modern obstetric care, and we have made massive progress towards making it available to every pregnant woman around the globe. Supply routes have been improved, and alternative packages like Uniject^®^ have been developed to try to make the logistics of injecting easier. But Torlini et al. show that the quality of oxytocin remains a problem in settings where the availability of fridges is limited and electricity supplies are patchy. In their review of published studies, over a third of samples had inadequate levels of oxytocin and over 45% failed quality tests.

How big a problem is this? Increasing the availability of oxytocin globally has been a central plank of safe motherhood strategy for at least the last 20 years, over which time maternal deaths have decreased by 45%. The increase in oxytocin availability was thought to have played an important role in this, but this is very difficult to quantify. For although numerous studies have shown that oxytocin prophylaxis reduces postnatal blood loss in women at low risk, it is questionable as to whether it actually prevents maternal death (Weeks et al. *BMJ* 2015;351:h3251). In settings with minimal health services, about one in 200 pregnancies will end in the mother's death from postpartum haemorrhage (PPH). But most of these are caused by uterine rupture or placental pathology (abruption, praevia or retained), for which oxytocics are relatively ineffective. Preventing maternal deaths from PPH globally will take more than oxytocin: providing safe blood transfusion and skilled surgery are likely to be far more important. The role of oxytocin in PPH is likely to be more about reducing the morbidity of postnatal anaemia than reducing maternal deaths.

Unfortunately there are also risks to intrapartum oxytocin. This is mainly linked to uterine hyperstimulation when oxytocin is used for induction and augmentation. But oxytocin infusions are also associated with retained placenta in a dose‐dependent fashion: 10 hours of oxytocin increases the risk of retained placenta by more than six times (Endler et al. *Obstet Gynecol* 2012;119:801–9). PPHs are common after a long augmented labour, but this may result from prolonged labour rather than from oxytocin exposure, as placebo‐controlled randomised trials of intrapartum oxytocin use show no increase in PPH.

The dangers multiply in settings where it is used indiscriminately. Intrapartum intramuscular oxytocin has become a common community treatment for slow labour in some parts of the world, with reports of unskilled health workers injecting oxytocin routinely to speed up labour. Overall, in these settings, intrapartum oxytocin infusions seem to double the number of perinatal deaths (Lovold et al. *Int J Gynaecol Obstet* 2008;103:276–82).

Increasing the quality and availability of oxytocin is certainly important for improving maternity outcomes worldwide, but only if used in the context of good‐quality care. If the issues of poor quality care are not simultaneously addressed, then it could easily cause more harm than good.

## Disclosure of interests

None declared. Completed disclosure of interests form available to view online as supporting information.
